# Supplementary material for: Enhancer-associated long non-coding RNA LEENE regulates endothelial nitric oxide synthase and endothelial function
Source: Nat Commun. 2018 Jan 18;9:292. doi: 10.1038/s41467-017-02113-y (PMC5773557; doi:10.1038/s41467-017-02113-y)
Supplement: Supplementary file 1 — Supplementary Information [file 41467_2017_2113_MOESM1_ESM.pdf]

Supplementary Figure 1

Subcellular quantitation for lncRNAs DANCR, Casc7, and TUG1

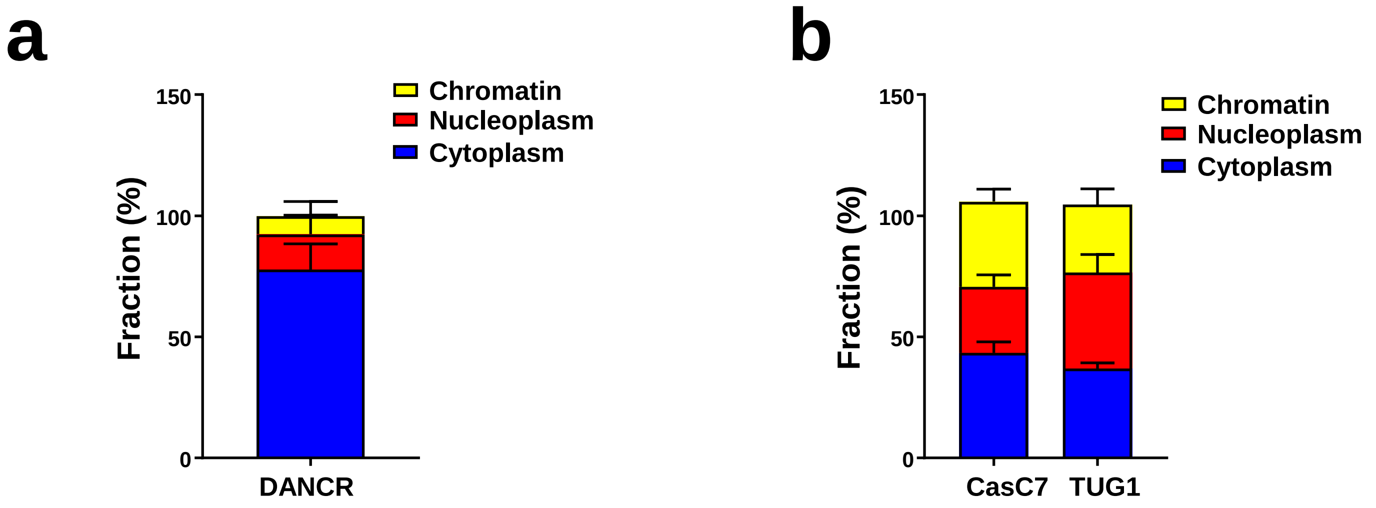

Supplementary Figure 1: qPCR quantitation of DANCR (a), CasC7, and TUG1 (b) in subcellular fractions from ECs, plotted as percentages in association with chromatin, nucleoplasm, and cytoplasm. Data are presented as mean±SEM. ANOVA followed by Bonferroni post-test is applied. n=5/group.

Supplementary Figure 2

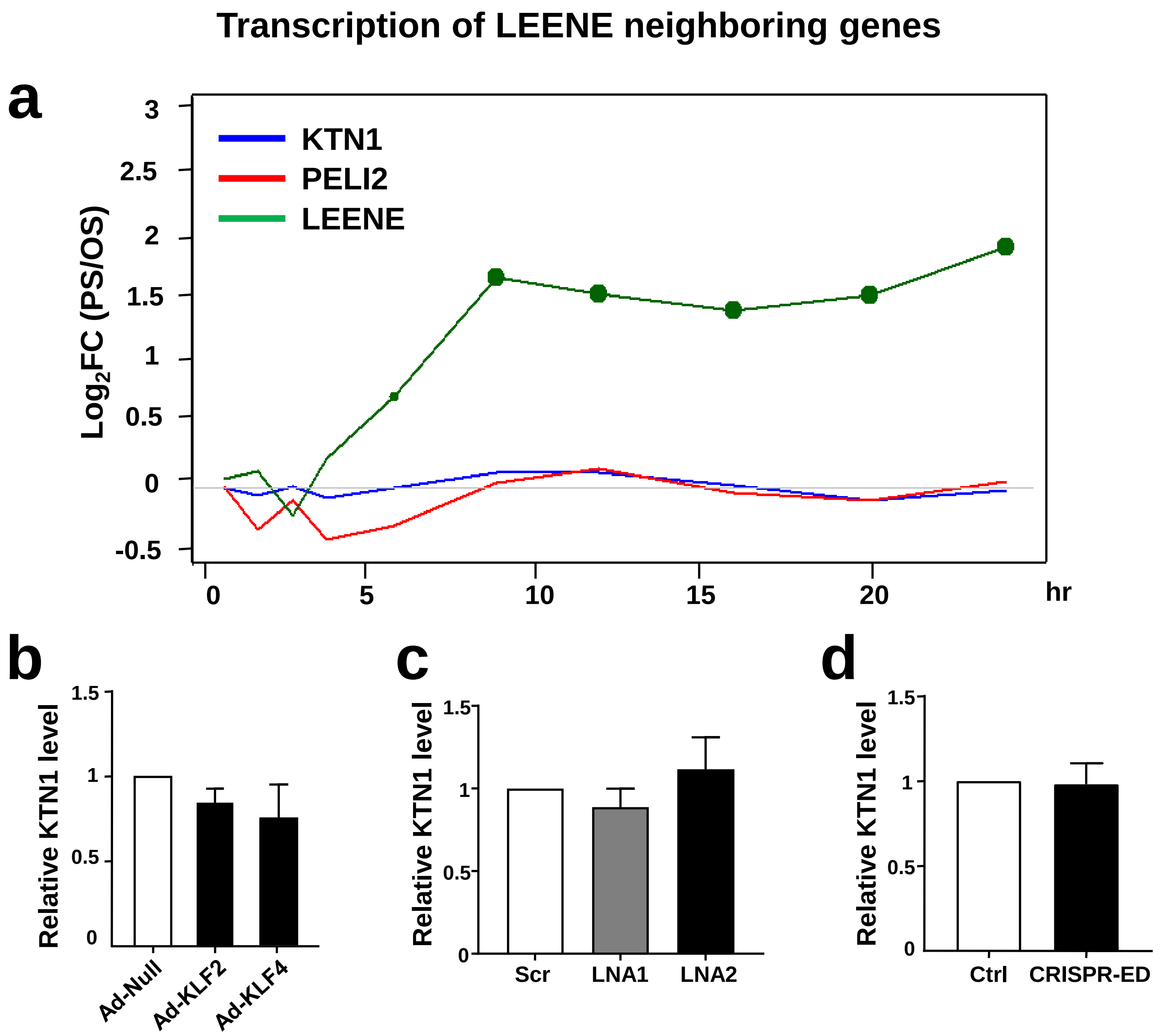

Supplementary Figure 2: (a) Time course of log_2FC (PS/OS) of LEENE and mRNAs encoding KTN1 and PELI2. (b-d) qPCR quantification of KTN1 in ECs with (b) KLF2 or KLF4 overexpression, (c) LEENE knock-down with two different gapmer-LNA (50 nM), or (d) CRISPR-cas9 gene editing targeting *LEENE* promoter/enhancer regions. Data are presented as mean±SEM. ANOVA followed by Bonferroni post-test is applied, n=5/group.

Supplementary Figure 3

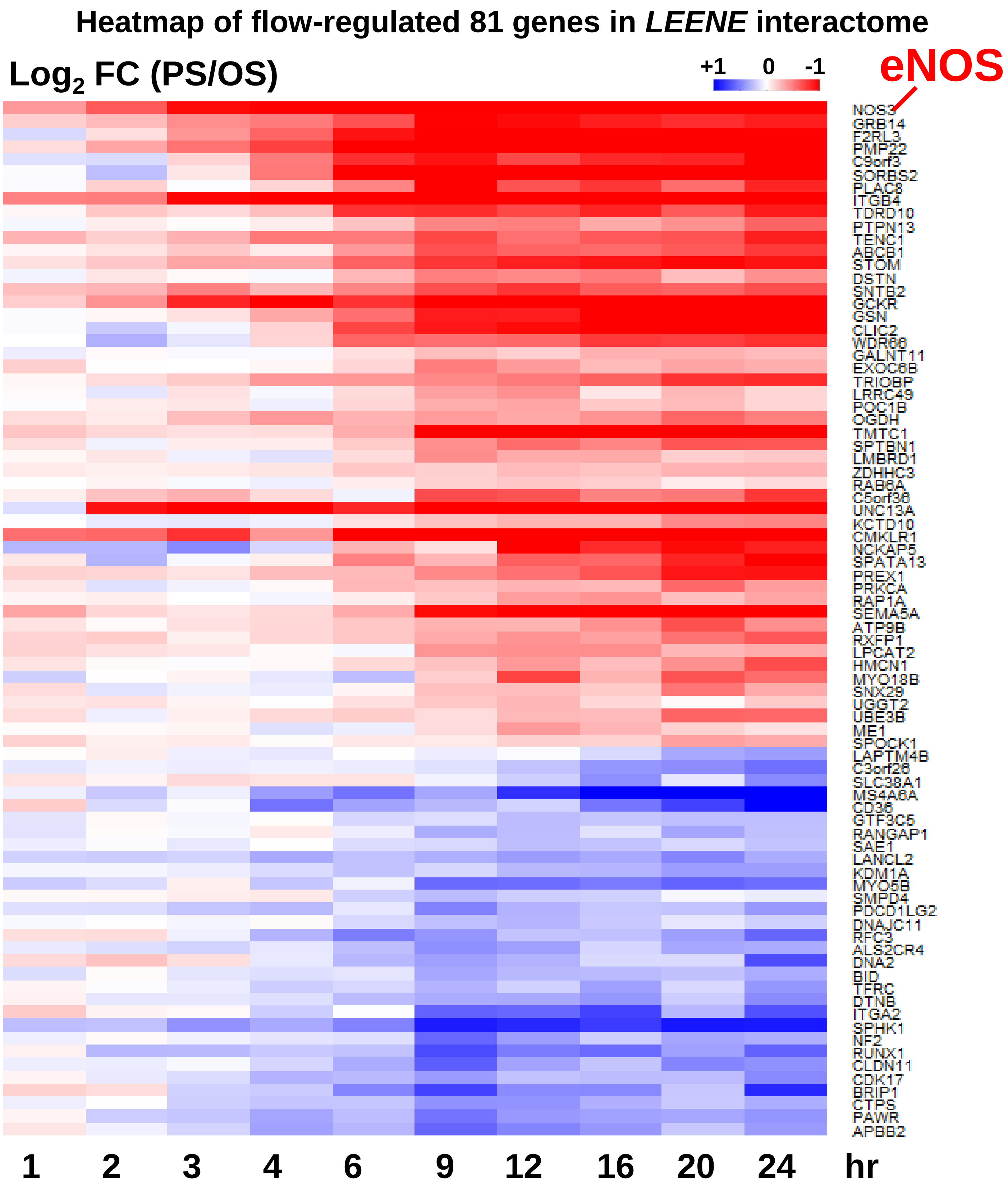

Supplementary Figure 3: Heatmap demonstrates the flow-regulated 81 genes in the *LEENE* interactome in Fig. 3d, with eNOS as one of the top correlated transcripts with LEENE.

Supplementary Figure 4

Hi-C analysis of *LEENE-eNOS* interaction map in non-EC cell types

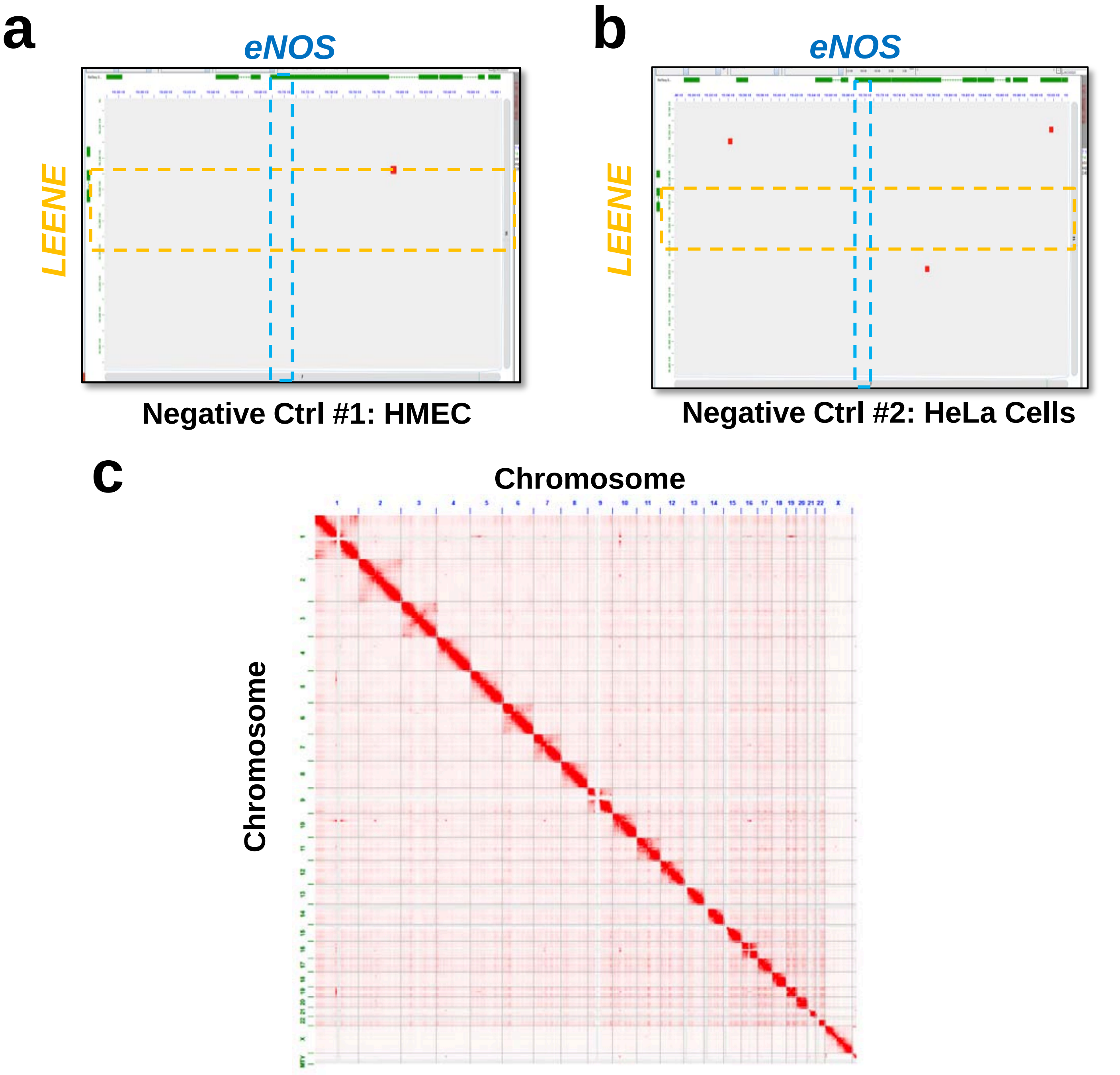

Supplementary Figure 4: *LEENE-eNOS* interaction map generated from Hi-C data from human mammary epithelial cells HMEC (a) or HeLa cells. Red pixels represent interactions between two regions respectively in chr 7 (X axis) and chr 14 (Y axis). The highlighted regions correspond to *eNOS* promoter and *LEENE* enhancer regions. Note that no red pixel was shown in the overlapped highlighted region. (c) A genome-wide contact matrix produced by Hi-C. Each pixel represents a chromosomal interaction using 5-kb windows; intensity corresponds to the total number of reads.

Supplementary Figure 5

Histone ChIP-seq tracks of *eNOS* promoter/enhancer region from four different cell types

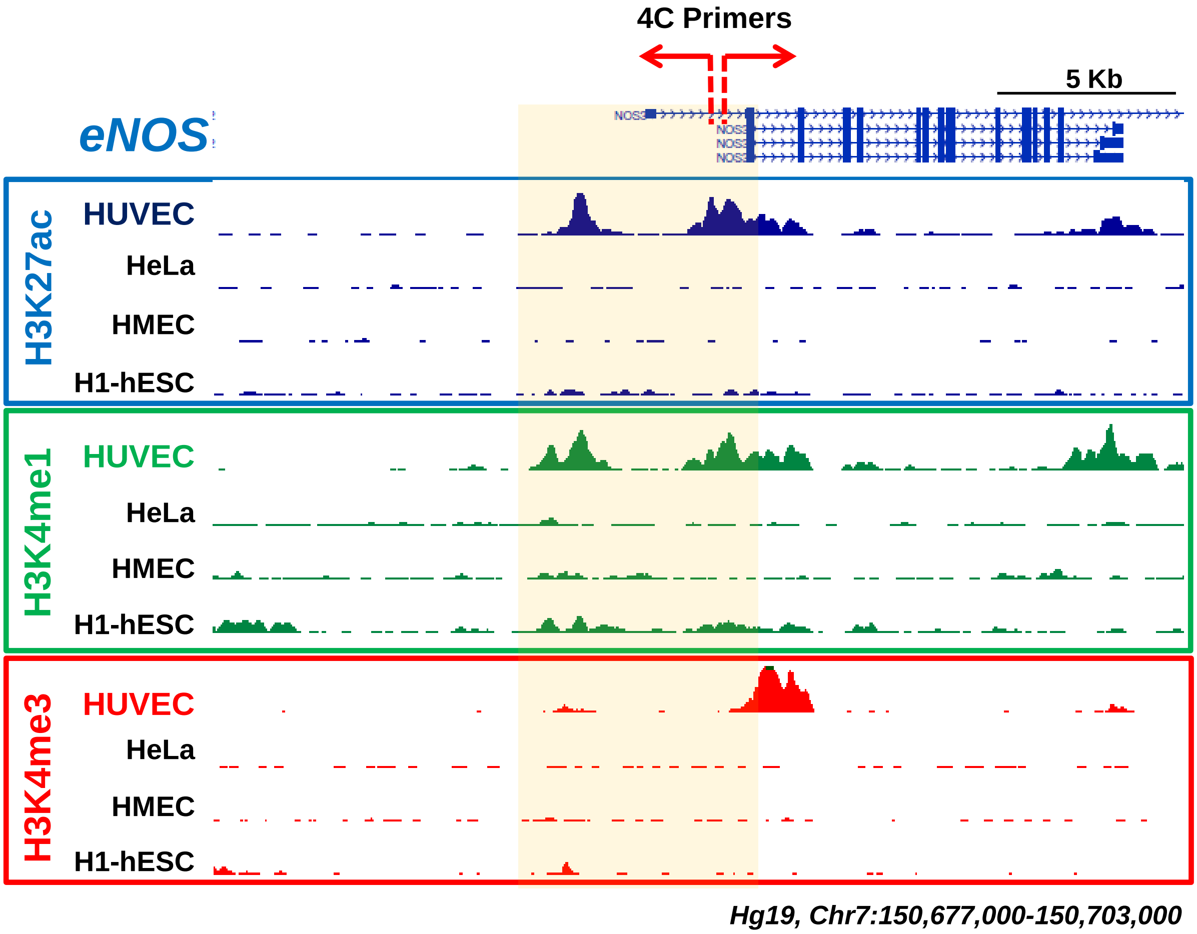

Supplementary Figure 5: ENCODE histone ChIP-seq signal from four different cell types surrounding *eNOS* promoter/enhancer region. Shaded area indicates *eNOS* promoter/enhancer regions. The red arrows indicate 4C library primers directions and locations. HMEC, human mammary epithelial cells; H1-hESC, human embryonic stem cells.

Supplementary Figure 6

Quality control of CRISPR-Cas9 gene editing

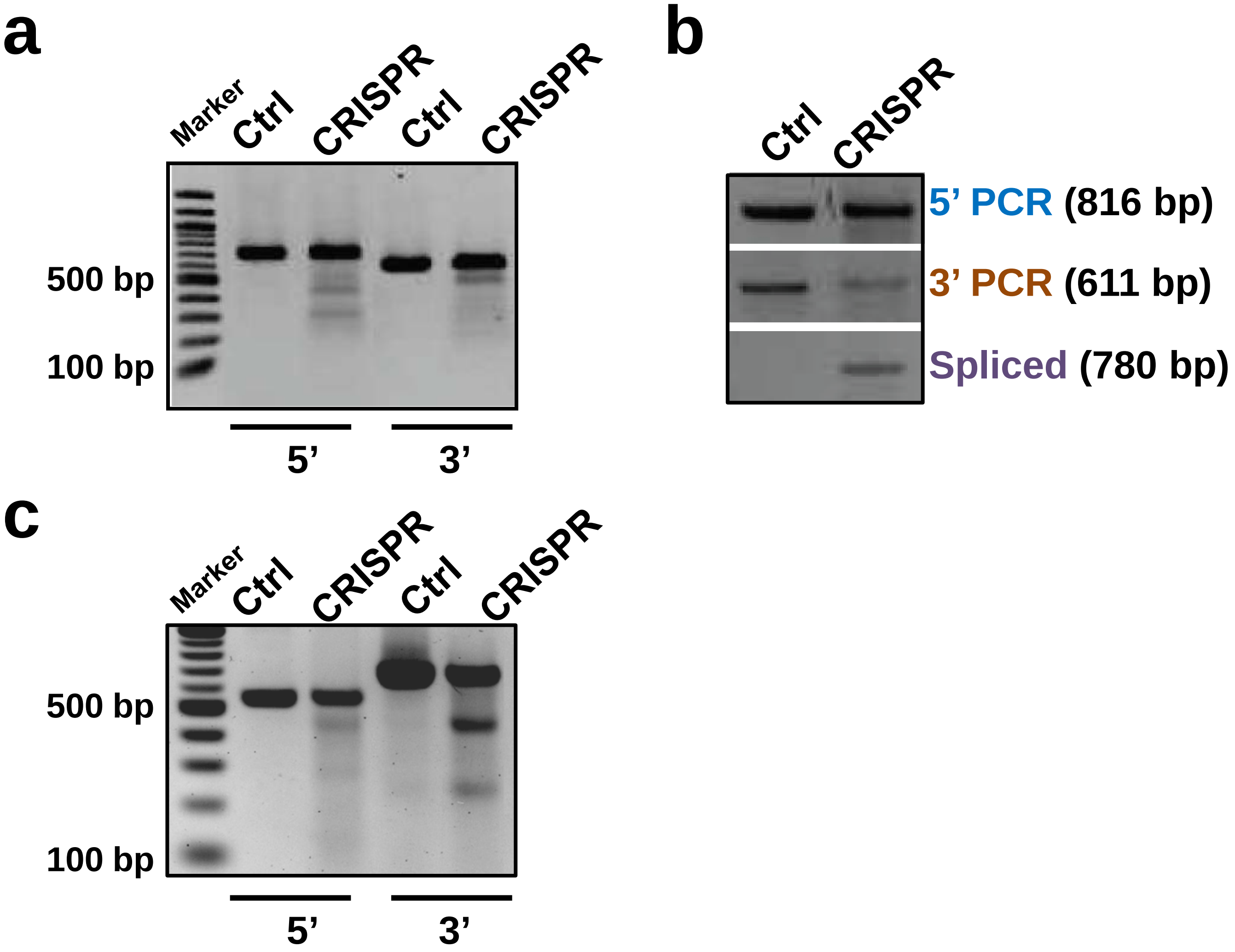

Supplementary Figure 6: Quality control of CRISPR-Cas9 gene editing system targeting *LEENE* promoter/enhancer (ED) (a and b) and coding region (CD) (c). (a) Surveyor assay for ED sgRNA-guided cas9 cutting efficacy in HEK293 cells. Select sgRNAs targeting respectively 5’ or 3’ ends were used to test the cutting efficiency as compared to control vector. (b) PCR validation of ED region of Cas9 targeting position as illustrated in Fig. 4a. The spliced and ligated 10 kb region is amplified in CRISPR-targeted but not control ECs. (c) Surveyor assay for CD sgRNA-guided cas9 cutting efficacy in HEK293 cells. Select sgRNAs targeting respectively 5’ or 3’ ends were used to test the cutting efficiency as compared to control vector.

Supplementary Figure 7

Effect of LEENE knock-down on eNOS mRNA level in HAoECs

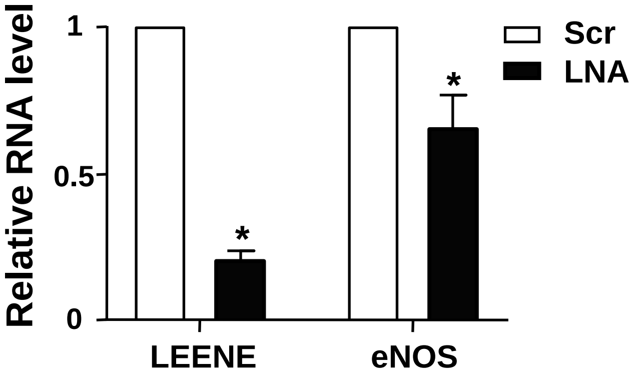

Supplementary Figure 7: Knock down of LEENE with LNA decreased mRNA expression of eNOS in HAoECs. Error bars represent mean±SEM, n=5/group. Student's *t*-test is applied, *p<0.05.

Supplementary Figure 8

Effect of LEENE knock-down on pro-inflammatory gene expression

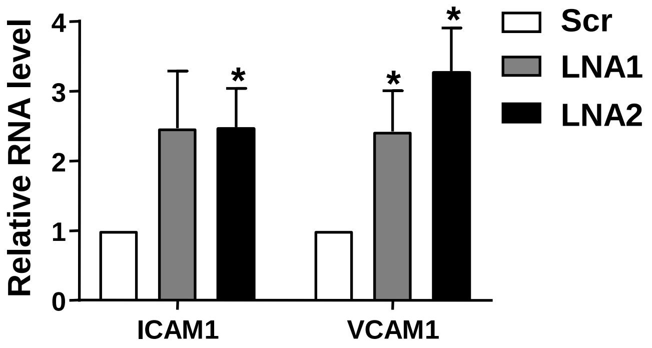

Supplementary Figure 8: Knockdown of LEENE with LNAs induced ICAM1 and VCAM1 mRNA levels in HUVECs. Error bars represent mean±SEM, n=5/group. ANOVA followed by Bonferroni post-test is applied, $*p<0.05$.

Supplementary Figure 9

Densitometry quantification of Western Blot experiments

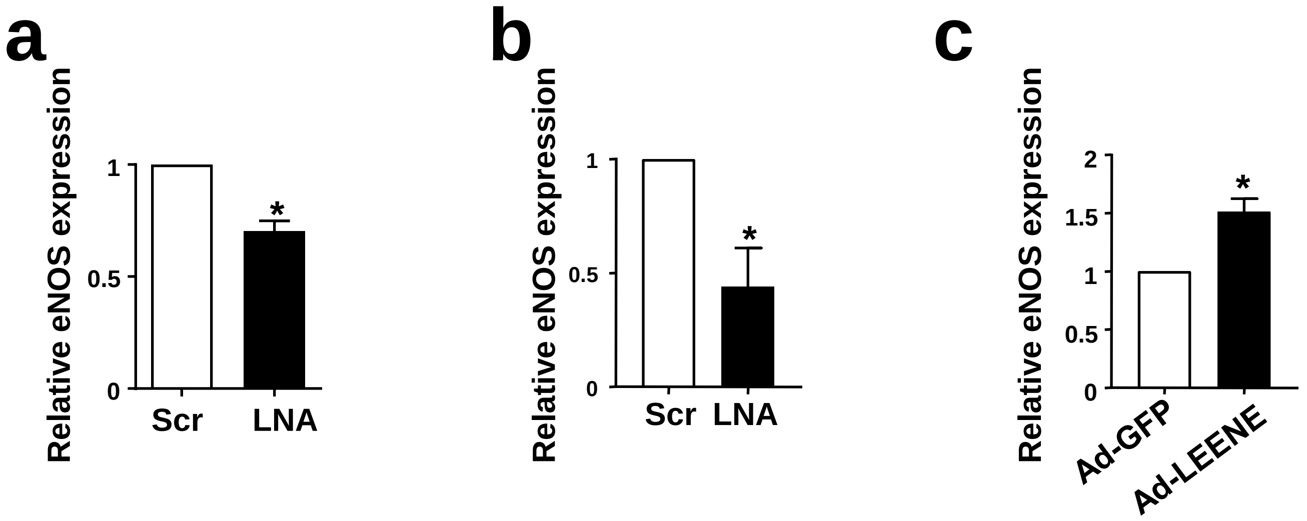

Supplementary Figure 9: a: Fig. 5b, under statin treatment; b: Fig. 5b, under PS condition; c: Fig. 5f. Error bars represent mean±SEM, n=5/group. Student's *t*-test is applied, *p<0.05.

Supplementary Figure 10

Overexpression of LEENE using adenovirus

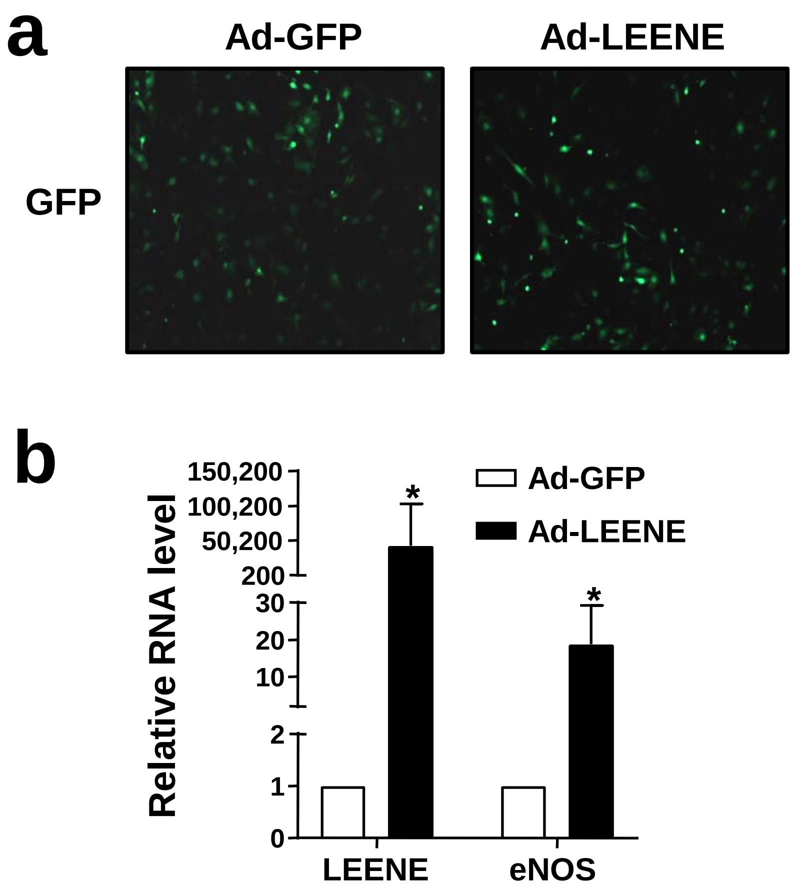

Supplementary Figure 10: (a) GFP images from HUVECs infected with Ad-GFP or Ad-LEENE adenovirus. (b) LEENE and eNOS RNA/mRNA in HAoEC infected with Ad-GFP or Ad-LEENE. Error bars represent mean±SEM, n=5/group. Student's *t*-test is applied, *p<0.05.

Supplementary Figure 11

IgG isotype control for the RIP assay

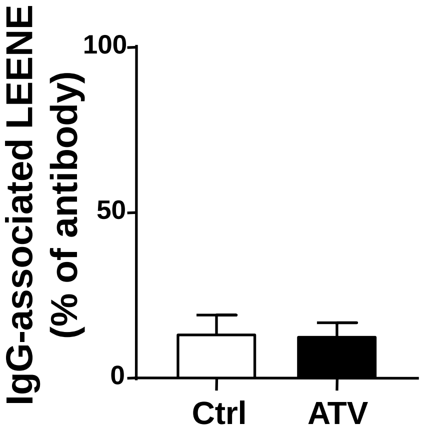

Supplementary Figure 11: Error bars represent mean±SEM, n=5/group. Student’s *t*-test is applied.

Supplementary Figure 12

Quality and negative controls of LEENE ChIRP assay.

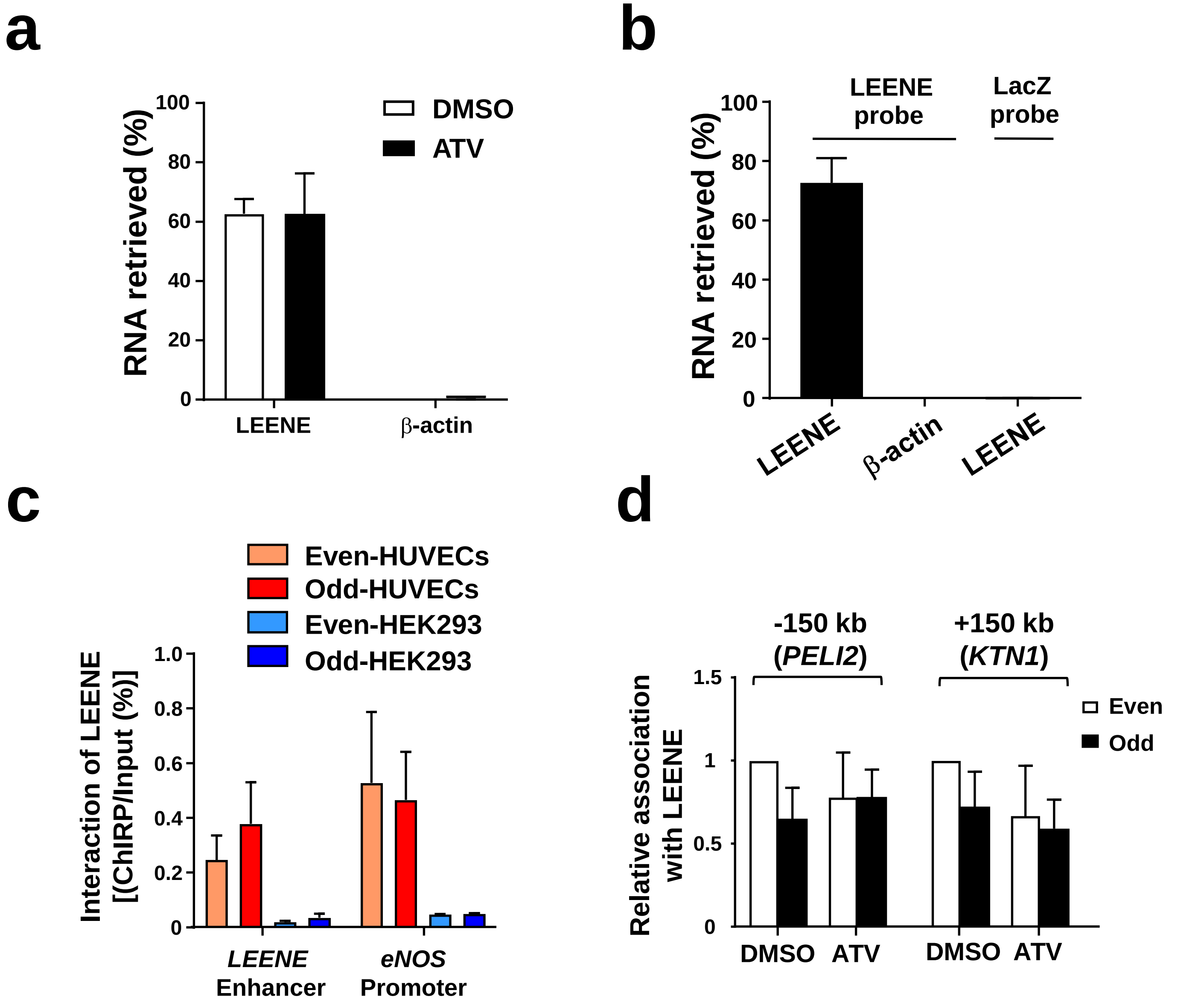

Supplementary Figure 12: (a) RNA retrieval rate using LEENE probe under DMSO or atorvastatin (ATV) treatment, quantified by qPCR. (b) RNA retrieval rate using LEENE or LacZ probe to pull-down LEENE RNA or β-actin under basal condition (DMSO). (c) LEENE ChIRP assay detecting LEENE RNA and *LEENE* enhancer/*eNOS* promoter region interactions using HUVECs or HEK293 cells. Error bars represent mean±SEM, n=5/group. (d) LEENE ChIRP qPCR detection of 150 kb up- an downstream regions respectively encoding PELI2 and KTN1. Error bars represent mean±SEM, n=5/group. ANOVA followed by Bonferroni post-test is applied.

Supplementary Figure 13

Effect of LEENE depletion on KLF4/Med1 binding to *eNOS* promoter

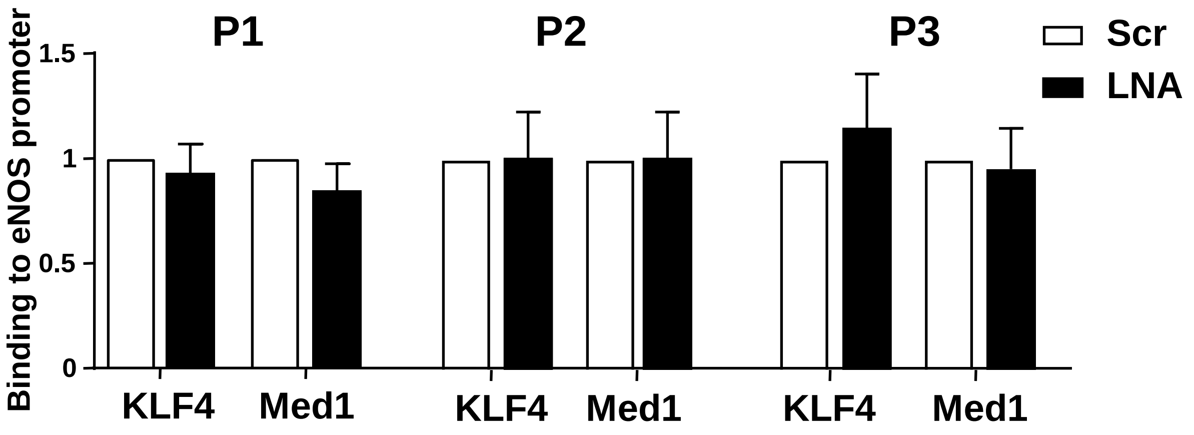

Supplementary Figure 13: KLF4 and Med1 were immunoprecipitated from ECs transfected with scramble LNA or LEENE LNA and their bindings to *eNOS* promoter region were detected by qPCR. Error bars represent mean±SEM, n=5/group. Student's *t*-test is applied.

Supplementary Figure 14

LEENE enhancer is prerequisite to LEENE RNA-*eNOS* locus association and LEENE RNA is not sufficient to enhance eNOS level

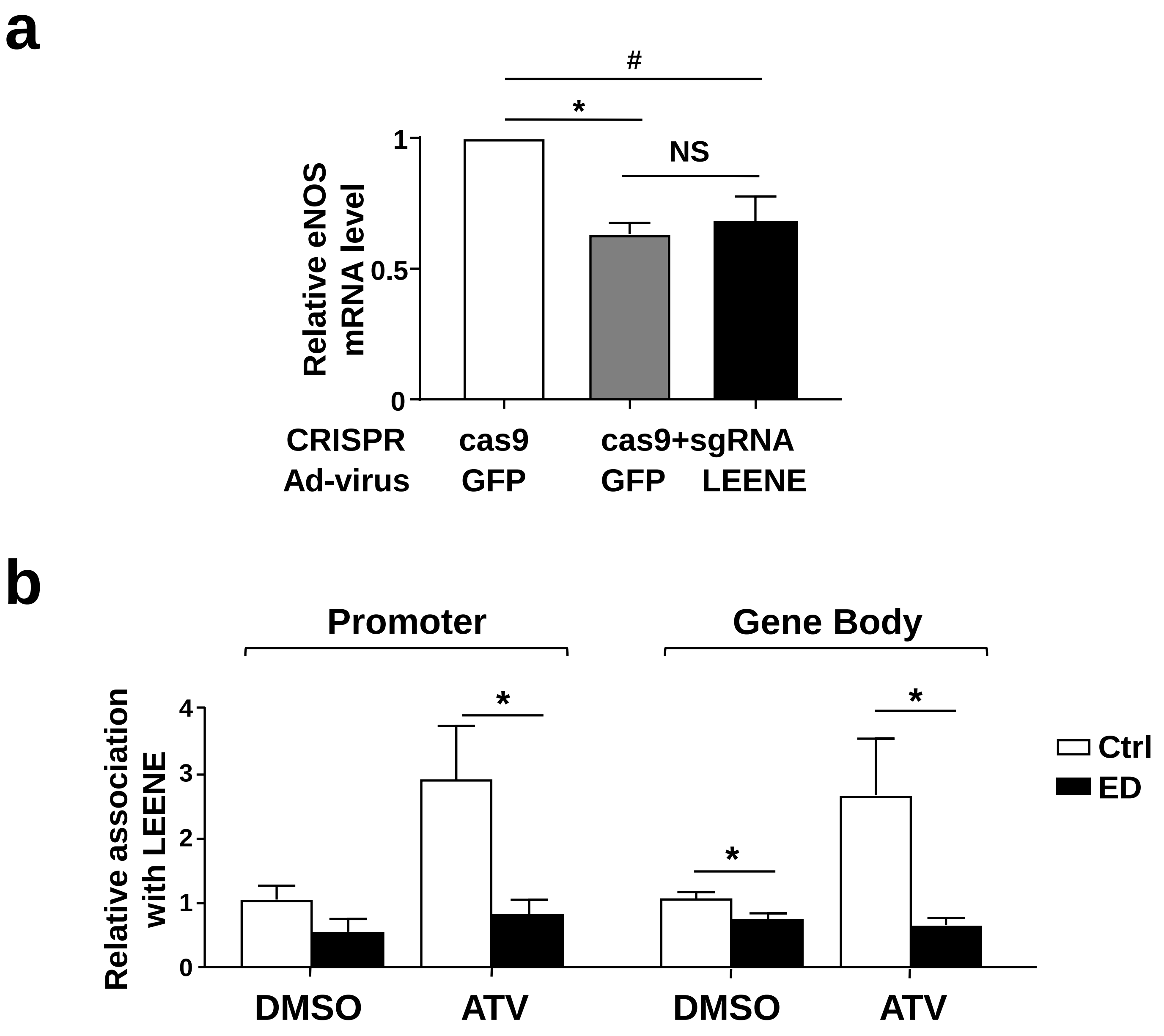

Supplementary Figure 14: (a) HUVECs were transfected with control (Ctrl) cas9 plasmid or Cas9-sgRNA targeting *LEENE* enhancer region (CRISPR-ED) 24 hr before infection with Ad-GFP or Ad-LEENE for another 48 hr. eNOS mRNA level was quantified by qPCR.(b) ChIRP detection of *eNOS* genomic locus binding with LEENE RNA in ECs transfected with Cas9 and sgRNA targeting the enhancer of *LEENE* and treated with DMSO or ATV. Error bars represent mean±SEM, n=5/group. ANOVA followed by Bonferroni post-test is applied, $*,^{\#}p<0.05$.

Supplementary Figure 15

Pathway enrichment analysis of *LEENE* interactome

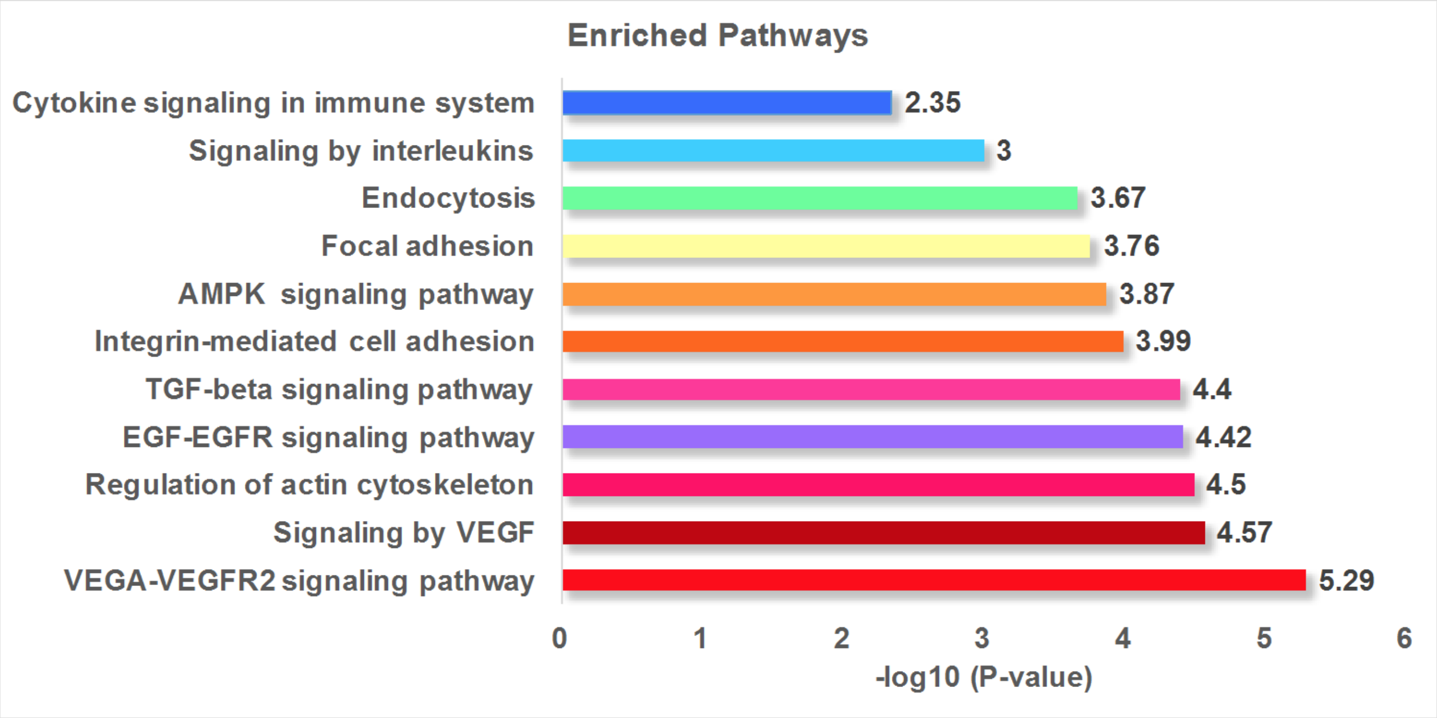

Supplementary Figure 15: Pathway enrichment analysis from 1177 genes that are differentially regulated by PS/OS and interact with *LEENE* genomic loci.

Supplementary Figure 16

Effect of LEENE knock-down or overexpression on Tm mRNA level

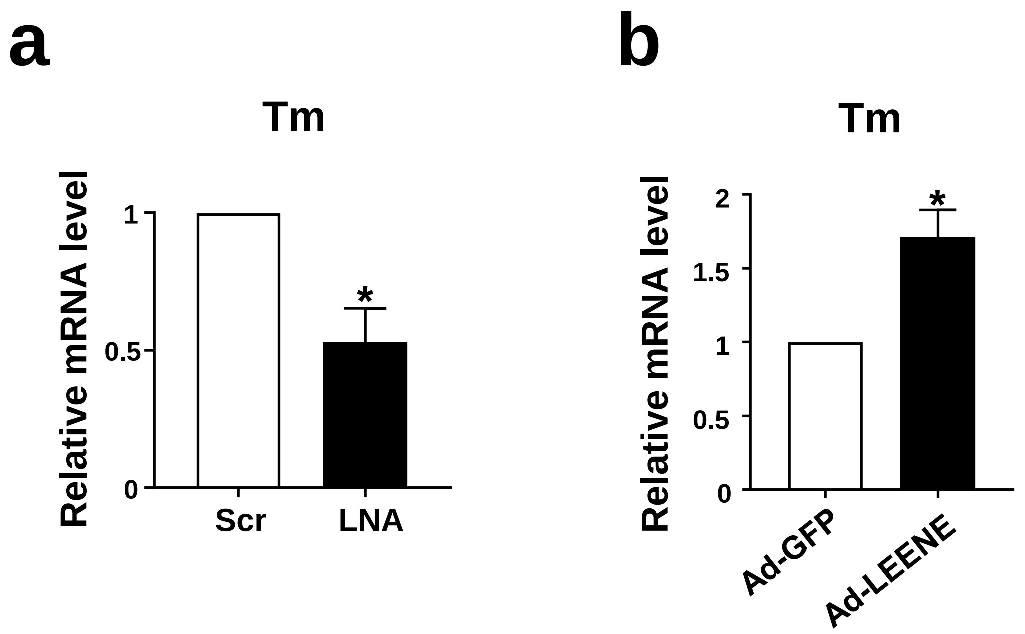

Supplementary Figure 16: Inhibition of LEENE by LNA decreased (a), while LEENE overexpression increased thrombomodulin mRNA level (b). Error bars represent mean±SEM, n=5/group. Student's *t*-test is applied, *$p<0.05$.

Supplementary Figure 17

Hi-C analysis of *LEENE-Tm* interaction map in HUVECs

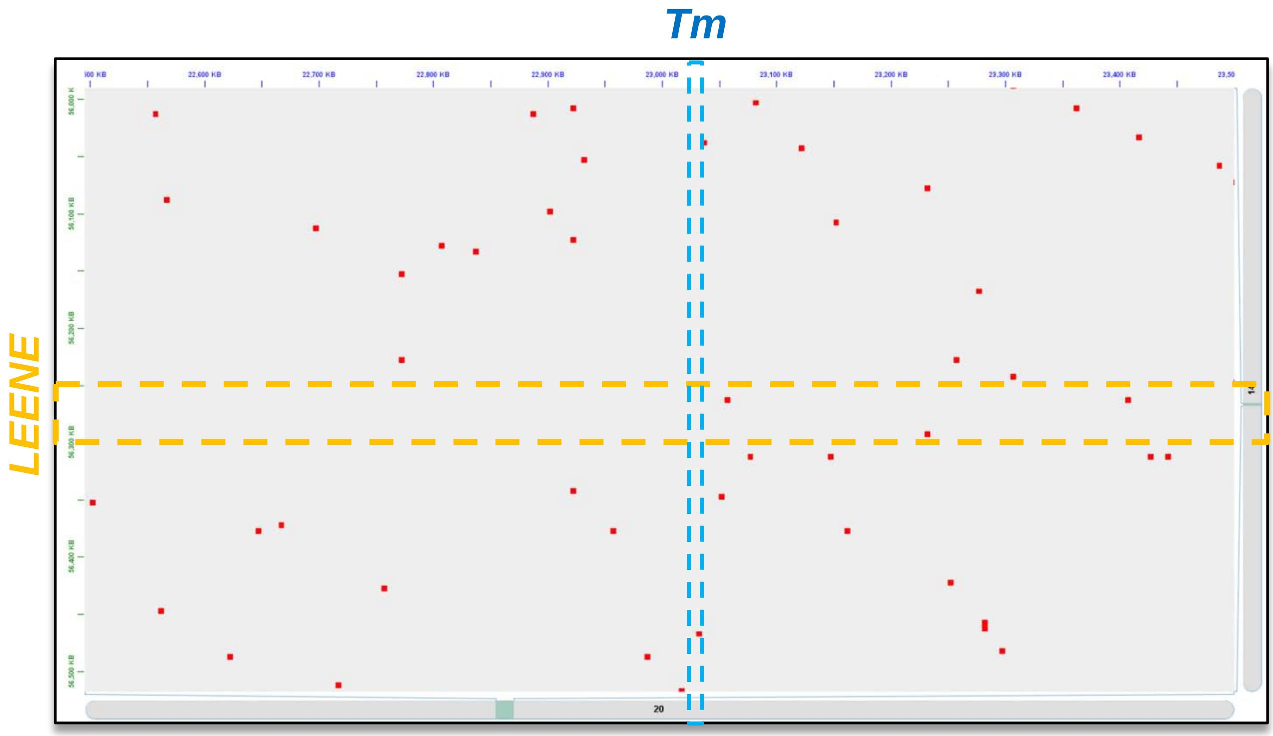

Supplementary Figure 17: *LEENE-Tm* interaction map generated from Hi-C data from HUVECs. Red pixels represent interactions between two regions respectively in chr 20 (X axis) and chr 14 (Y axis). The highlighted regions correspond to *Tm* promoter and *LEENE* enhancer regions. Note that no red pixel was shown in the overlapped highlighted region.

Supplementary Figure 18

Detailed gene information of 4C-seq circoplot

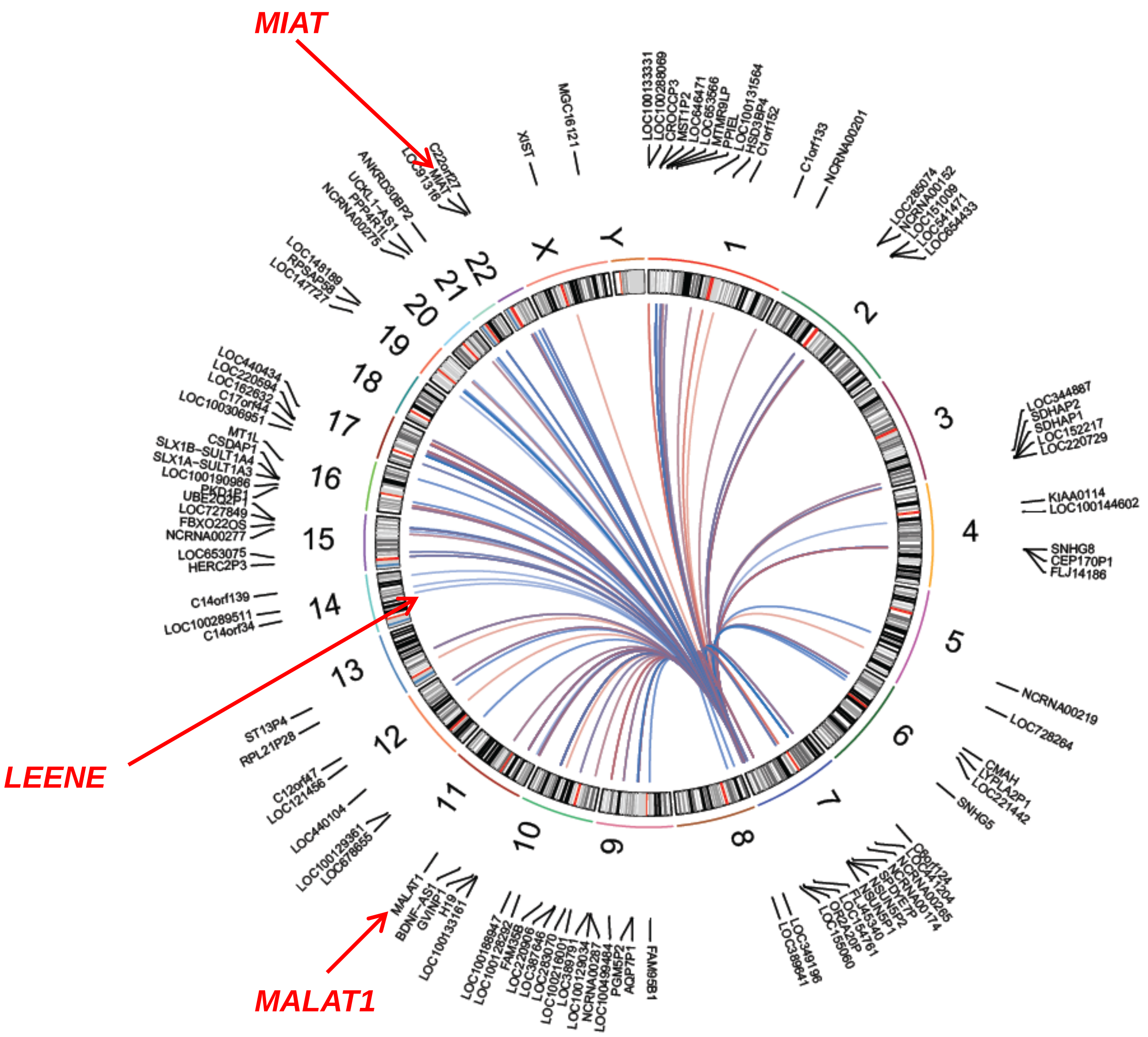

Supplementary Figure 18: Detailed gene information of Fig. 3g. Each line in circoplot depicts a chromosomal interaction between lncRNAs (identified in Fig. 1b) and eNOS bait as revealed by 4C-seq. Blue lines: PS; red lines: OS.

Supplementary Figure 19

Original uncropped scan of western blots

Fig. 5b

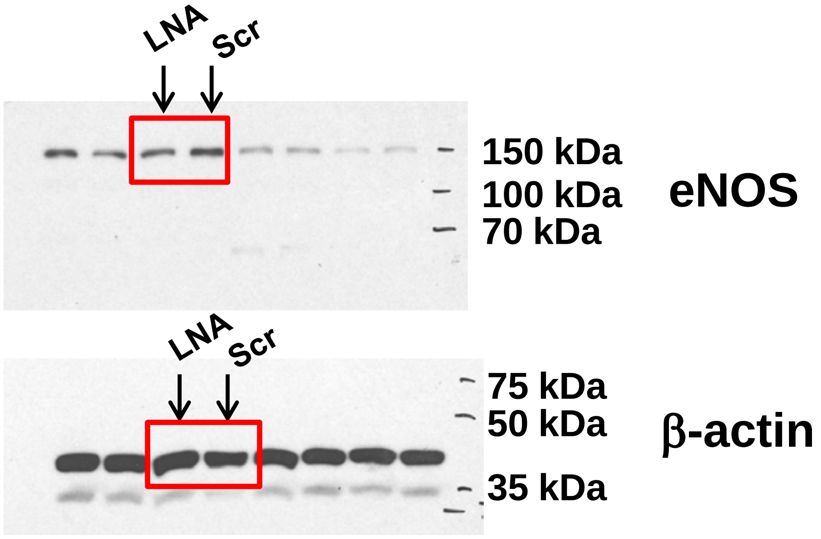

Supplementary Figure 19: Original uncropped scan of blots for Fig. 5b, left side 'ATV'

Supplementary Figure 20

Original uncropped scan of western blots

Fig. 5b

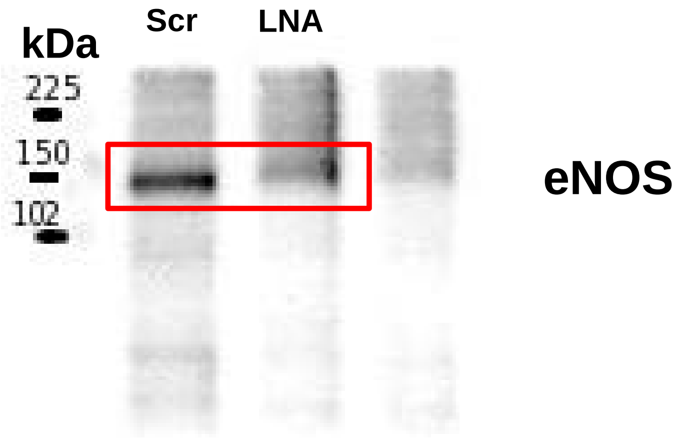

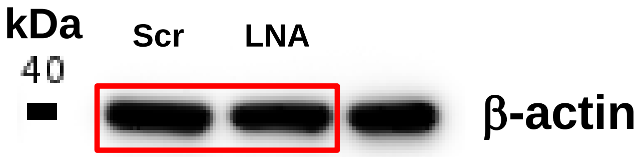

Supplementary Figure 20: Original uncropped scan of blots for Fig. 5b, right side 'PS'

Supplementary Figure 21

Original uncropped scan of western blots

Fig. 5f

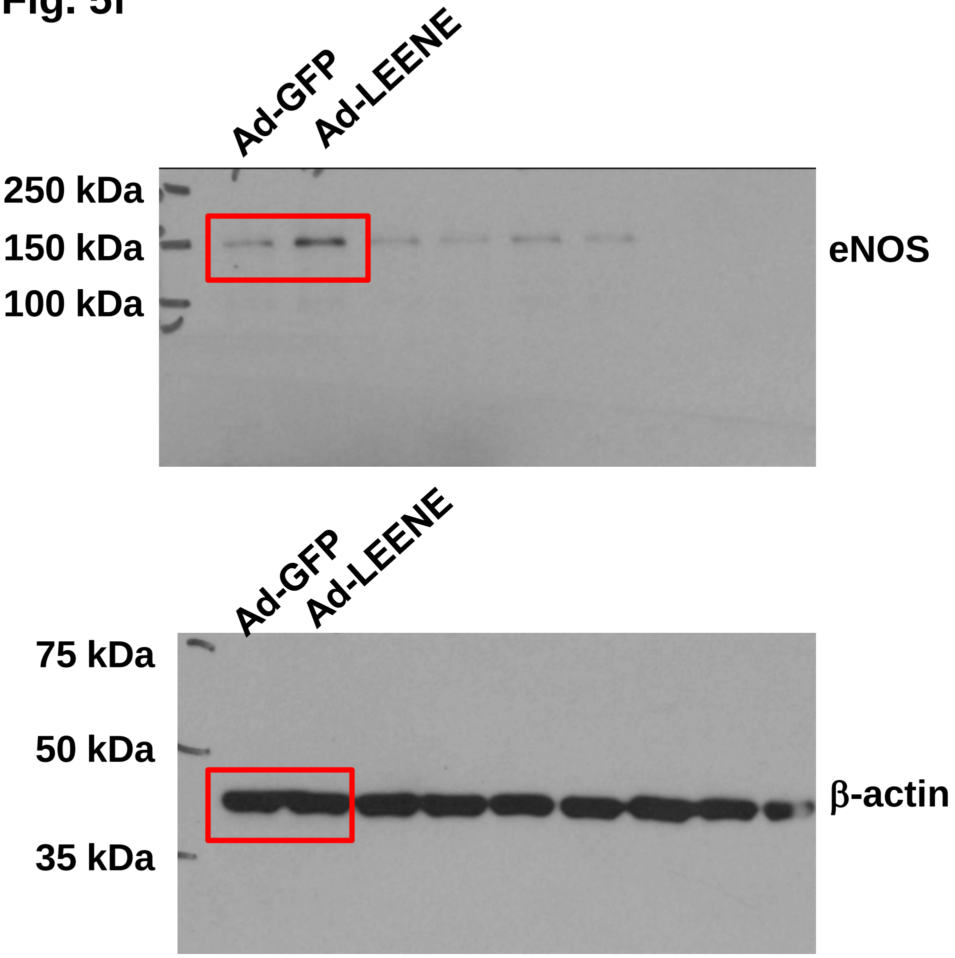

Supplementary Figure 21: Original uncropped scan of blots for Fig. 5f

Supplementary Table 1 Human primer sequences

Name (Human)	Forward	Reverse
**qPCR primer**		
LINC00520 (LEENE)	TTTCCCTCTTTGGGGTCTCA	GCCCTTTGATGAGTGAGTCG
eNOS	TGATGGCGAAGCGAGTGAAG	ACTCATCCATACACAGGACCC
KLF2	CATGTGCCGTTTCATGTGC	GCAAGACCTACACCAAGAGTTCG
KLF4	CCCACATGAAGCGACTTCCC	CAGGTCCAGGAGATCGTTGAA
VCAM1	GTCAATGTTGCCCCCAGAGA	TTTTCGGAGCAGGAAAGCCC
ICAM1	GTGTCCTGTATGGCCCCCGACT	ACCTTGCGGGTGACCTCCCC
ACTB	CATGTACGTTGCTATCCAGGC	CTCCTTAATGTCACGCACGAT
MALAT1	GTGCTACACAGAAGTGGATTC	CCTCAGTCCTAGCTTCATCA
DANCR	AGGAGTTCGTCTCTTACGTCT	TGAAATACCAGCAACAGGACA
CasC7	GAGGCTGACCTAAACTGCTATC	CCACTACAGGAACTGAACTGAC
TUG1	CTGTGACCCAGAAGAGTTAAG	CATATCCCAGGGACTCAAAC
KTN1	AAGGAAAGGCAGCAACAGGT	CTGACCCTGAAGTTCCAGCC
Tm	ACCTTCCTCAATGCCAGTCAG	CGTCGCCGTTCAGTAGCAA
**4C primer**		
eNOS 4C Primer	AGGACTCAAGGGTGGGGATC	GCAGGTCAGCAGAGAGACTA
**H3K27ac ChIP-qPCR primer**		
Region I	GGCCAATGCATTTGACCTCC	GCTTGTTGGCCACCTGATTT
Region II	CGGTCACTTGCCTGACATCT	GGGCACAGTGATCCACTTCA
Region III	ACTGACACTGAGCTCTCCCT	CCCAGCCGGGATTTACCTTT
Region IV	ACCTTTGCTACCGGAACTCG	GAGAGAGAACGTGACGTGGG
**ChIP-qPCR primer**		
**KLF4 ChIP**		
eNOS Promoter	GCCCAGCAAGGATGTAGTGA	GTGTGGGGGTTCCAGGAAAT
LEENE P1	CAGCCTCGTACACAGAAGCT	AACTCATTCCATCTTTAACCTGC
LEENE P2	CTGGTTTCAAACTCGTGGCT	AACGTAGATAGGAGGCCAGG
LEENE P3	GATAGGGCCTGGTGAATTGC	TTATGGAAGGAAGGGCAGGG
LEENE P4	CCATGGCTTTGAAACTGCC	GCAAAGACAACCACCCTGTA
**Pol II, KLF4, and Med1 ChIP**		
eNOS Promoter P1	ATCCCCTGCTACAGAAACGG	AGCCACCAGGGGGTCATAAA
eNOS Promoter P2	GCCCAGCAAGGATGTAGTGA	GTGTGGGGGTTCCAGGAAAT
eNOS Promoter P3	GCCGAACACCAAATCTCCAAC	AGCCCTGCCAAGAATGATGC
**ChIRP-qPCR primer**		
LEENE Enhancer	CAGCCCTCACATGTATCCCC	GTGCTATGGAAGGTGGCGTA
eNOS Promoter	GCCGAACACCAAATCTCCAAC	AGCCCTGCCAAGAATGATGC
eNOS Gene Body	AACACATAGGCCCTGATTGGG	TGATGATGTGGTCTCATCCCG
150 Upstream (*KTN1*)	AGAGGTGATAGTGAAGAATGACT	CAGAAGACAGGTGAAAGGGT
150 Downstream (*PEIL2*)	GAAAGAGGCTGTGAGGATGA	TTTCAACTCAGCTGCTATGC
**CRISPR PCR primer**		
5' site	CAATGATAGGCTGGATTAAGAAAATGTGGC	GATGTTCTGATAAAATCATTGCAATCTGGGC
3' site	TGTCATGGGAGGGACTGAATGG	GGCCACATATTGTATGATTCCATTTATATGAG ATG
Spliced region	CAATGATAGGCTGGATTAAGAAAATGTGGC	GGCCACATATTGTATGATTCCATTTATATGAG ATG

Supplementary Table 2 Mouse primer sequences

Name (Mouse)	Forward	Reverse
**qPCR primer**		
BY707159.1 EST	GGACCTCTGGCTAAGGTGAG	TCCTTGCTTGTCCTGTCAGT
eNOS	CACCTACGACACCCTCAGTG	CTTGACCCAATAGCTGCTCAG

Supplementary Table 3 sgRNA sequences

sgRNA name	Sequence
**ED_5’-1**	ATGGGTGCAACACACCAACA
**ED_5’-3**	GGTGGGGAACATCACACACT
**ED_5’-6**	GAGGGGGGAGGGATAGCATT
**ED_3’-7**	GTCATATCTTACATGGCAGA
**ED_3’-9**	GAGCACAGTCATATCTTACA
**CD_5’-1**	TACCATAACACTAGACCAGC
**CD_5’-2**	GCTCTCAGTCATTATATGAT
**CD_5’-3**	AGTAAAATCACCCCGTGTAG
**CD_3’-1**	GAAACCCCGAGCCAAACGTA
**CD_3’-2**	TGTGTTACGGTCTTCAATGG
**CD_3’-3**	CAGCCTCCATTGAAGACCGT

Supplementary Table 4 LEENE LNA-GapmeRs sequences

	Sequence	Position (NR_026797)
**LNA-1**	TTTGATGAGTGAGTCG	218-233
**LNA-2**	GTTACGGTCTTCAATG	1628-1643
**Scramble**	AACACGTCTATACGC	

Supplementary Table 5 LEENE ChIRP probe sequences

Number	Sequence	Position
**1**	ATAGAATCTTGCTTGGGCAG	170-198
**2**	CTGAGTGGATTGTAGGTGTT	443-462
**3**	TCCTGTCTTCTTACTTGTAC	629-648
**4**	CCCCAAAATCCTTTAAGGTA	927-946
**5**	TGTCTCTGGGAAGAGGAGAG	1072-1091
**6**	TGGATGTAAAGACTGGTGCC	1173-1192
**7**	CTGAGCTGTAGAATCCACAG	1277-1296
**8**	CTGACATCTCATCAAGGGAG	1377-1396
**9**	GCTCATCAAGAAGCAGCTAG	1488-1507
**10**	AGTCACAAGAAGTCCAAGGC	1594-1613
